# MISMATCH BETWEEN PERCEIVED AND OBJECTIVE MOTOR IMPROVEMENTS WITH ADAPTED TANGO INTERVENTION IN OLDER ADULTS

**DOI:** 10.1093/geroni/igz038.605

**Published:** 2019-11-08

**Authors:** Crystal Bennett, Nathalie Angel, Madeleine Hackney

**Affiliations:** 1 University of West Florida, Pensacola, Florida, United States; 2 Emory University, Atlanta, Georgia, United States

## Abstract

The purpose of this secondary analysis was to assess the relationship between objective and subjective perceptions of motor function measures in older adults following a 12 week adapted tango or health education intervention. A quasi-experimental, two-group, repeated-measures design was used. The study took place in diverse senior independent living communities in an urban metropolitan area. 74 older adults participated (Tango: n= 62, age: 82.3 (8.81) years; Education: n=12, age: 84.1 (7.86) years). Participants were assigned to 20 sessions of 90-minute tango (n = 62) or health education (n = 12) classes over 12 weeks. Motor function, depression, mental and physical quality of life were measured before and after intervention. At post-test, participants indicated their level of agreement with statements that they improved in the objectively measured domains of motor function. Correlations were performed between subjectively rated agreement, and changes in motor function and depression/quality of life. Tango subjective ratings were negatively correlated with empirically observed improvements in balance (r= -.423) and endurance (r= -.241); although their ratings moderately correlated positively with coordination (r=.319) and minimally correlated positively with lower body strength (r=.188). In Tango, decreased depression was positively correlated with self-perceived improved lower body strength (r=.271) and endurance (r= .254). Improved mental function was moderately (r=.423) positively correlated with self-perceived improved balance and coordination (r=.306). After rehabilitation, even in the presence of improved depression and quality of life, older adults may not perceive empirically observed motor function improvements, particularly in balance and lower body strength.

